# Clone-advisor: recommending code tokens and clone methods with deep learning and information retrieval

**DOI:** 10.7717/peerj-cs.737

**Published:** 2021-11-09

**Authors:** Muhammad Hammad, Önder Babur, Hamid Abdul Basit, Mark van den Brand

**Affiliations:** 1 Eindhoven University of Technology, Eindhoven, Netherlands; 2 Wageningen University and Research, Wageningen, Netherlands; 3 Prince Sultan University, Riyadh, Saudi Arabia

**Keywords:** Language modeling, Deep learning, Code clone, Code prediction, Information retrieval, Code search

## Abstract

Software developers frequently reuse source code from repositories as it saves development time and effort. Code clones (similar code fragments) accumulated in these repositories represent often repeated functionalities and are candidates for reuse in an exploratory or rapid development. To facilitate code clone reuse, we previously presented DeepClone, a novel deep learning approach for modeling code clones along with non-cloned code to predict the next set of tokens (possibly a complete clone method body) based on the code written so far. The probabilistic nature of language modeling, however, can lead to code output with minor syntax or logic errors. To resolve this, we propose a novel approach called Clone-Advisor. We apply an information retrieval technique on top of DeepClone output to recommend real clone methods closely matching the predicted clone method, thus improving the original output by DeepClone. In this paper we have discussed and refined our previous work on DeepClone in much more detail. Moreover, we have quantitatively evaluated the performance and effectiveness of Clone-Advisor in clone method recommendation.

## Introduction

Software developers need effective code search and reuse capability for rapid or exploratory development (Sadowski, Stolee & Elbaum, 2015), as writing source code from scratch is an expensive activity. Often, programming of well-defined features amounts to a simple look-up in one’s own or others’ code in repositories. With the increasing volume of available source code repositories and online resources, it gets more probable to find useful code snippets. Nevertheless, for identifying the relevant parts of the code for reuse, developers turn to ad-hoc code reuse with manual searching and selective reading of the source code (Gharehyazie, Ray & Filkov, 2017). It is an expensive and error-prone activity, if not effectively supported by automated mechanisms like code snippet search, code prediction, code auto-completion and code generation. Language modeling is amongst the most popular methods to realize these features (Radford et al., 2019b; Karampatsis et al., 2020; Zhong, Yang & Sun, 2019).

Shannon first introduced language modeling (Shannon, 1948; Shannon, 1951) to predict the next element following some given text, and bound the entropy of the English language. Several language modeling techniques (Allamanis & Sutton, 2013, Boldt, 2017; Hellendoorn & Devanbu, 2017; White et al., 2016) have since been developed to perform different tasks. A language model (LM) estimates the likelihood of sequences of tokens based on a training dataset, by assigning probabilities to tokens (words, subwords, or punctuation marks) or character sequences (sentences or words occurring after a given sequence (Jurafsky & James, 2009)). Different statistical and deep neural networks (DNN) based techniques have been applied for LMs (Allamanis & Sutton, 2013; Boldt, 2017; Hellendoorn & Devanbu, 2017; White et al., 2016). Both types of techniques have led to great results in natural language processing (NLP) tasks, as natural language is often repetitive and predictable (Hindle et al., 2016), thus can be modeled using either of the techniques. However, statistical modeling techniques do not handle large vocabularies very well. Source code is an example of a language with large vocabulary as developers frequently declare new identifier names, which degrades the performance of statistical language models on source code tasks (Karampatsis et al., 2020).

DNNs are extremely powerful machine learning models that achieve excellent performance on various difficult problems such as speech recognition (Dahl et al., 2011) and visual object recognition (Liu et al., 2020). A recent study (Karampatsis et al., 2020) shows that DNNs indeed outperform statistical modeling techniques in language modeling for source code. Their power arises from the fact that they can perform arbitrary parallel computation for a modest number of steps. LMs built using DNNs are referred as Neural Language Models (NLM). NLMs have been used to perform various tasks such as machine translation (Luong, Kayser & Manning, 2015), comment generation (Hu et al., 2018), code completion (Mou et al., 2015), code clone detection (White et al., 2016), code search (Gu, Zhang & Kim, 2018) and code summarization (Iyer et al., 2016).

A popular application of language modelling is code prediction (Karampatsis et al., 2020; White et al., 2015; Boldt, 2017). It involves automating software development and maintenance by proposing next likely tokens based on user input. A prediction model is capable of automatically learning features for representing source code, and using them for next token prediction in a sequence. As a notable example, Pythia (Svyatkovskiy et al., 2019) is a code completion system trained on source code snippets by using Long Short Term Memory (LSTM), which predicts ranked lists of method and API recommendations at edit time. Similarly, Deep TabNine (https://www.tabnine.com/) is a recently launched auto-complete tool fine-tuned by using GPT-2 on approximately two million GitHub files and aims to enhance software developers’ workflows. GPT-2 (Radford et al., 2019a; Radford et al., 2018; Radford et al., 2019b) is a large transformer-based language model with 1.5 billion parameters, trained on a dataset of eight million web pages. GPT-2 is trained with a simple objective: predict the next word, given all of the previous words within some text.

In previous work, we introduced DeepClone (Hammad et al., 2020a), a DNN model trained by fine-tuning GPT-2 over the BigCloneBench code clone dataset, for predicting code tokens and clone methods. The proposed approach has already led to promising results. The performance metrics in the training (learning rate approaching 0, minimized loss) and validation (perplexity of 2.145) phases indicate a fine-tuned model. The series of perplexity scores calculated allow us to conclude that DeepClone model can predict regularities successfully in terms of clone markers, including the code in general and the individual clone method predictions in particular. The extrinsic evaluation reveals that we achieve high accuracy, notably 95% in the top ten suggestions, as well as larger number of tokens than a threshold-based strategy even with a generous threshold of 100. DeepClone model can assist developers in predicting the next token (as typically done by many language models) or the complete clone method body.

DeepClone, despite the promising results, has a shortcoming. It predicts clone methods that differ from the real clone methods because of the limitation of language models, and the specific neural language generation technique applied (nucleus sampling (Holtzman et al., 2019)). This can lead to various syntax or logic errors. For instance, in Table S1, “destDir” identifier has been declared in the DeepClone output, but it has not been used anywhere. This problem is not specific to DeepClone but is a well-known challenge in natural language models to predict well-formed outputs (Li et al., 2017a; Shao et al., 2017). Language models (in our context) are fundamentally probabilistic models, which can predict multiple possible sequences of output (in our case predicted clone methods) based on user context. The space of possible methods that could be predicted grows exponentially with the length of these methods. By having *V* tokens in the vocabulary, there can be *V*^*N*^ possible methods of length *N* that could be predicted. A fully trained language model can learn patterns in the code such as opening and closing brackets, but it cannot completely learn the logical flow of the code.

While recently there are significant advancements in neural language generation techniques, they still cannot match the quality of human authored content (*e.g.*, programs or texts) (Fan, Lewis & Dauphin, 2018). They further possess certain problems at their core. For instance, standard likelihood training and decoding leading to dull and repetitive outputs (Holtzman et al., 2019). Moreover, more training data and advanced sampling techniques do not seem to solve this issue entirely (Radford et al., 2019b). Token-level probabilities predicted by the language models also remain relatively poor (Welleck et al., 2019). However, the desired output might be a variation of another, previously observed sample (Song et al., 2016; Hashimoto et al., 2018), which is elaborated next. This motivates our work here, where we seek to build a system that can recommend real clone methods based on predicted clone method. Here, a real clone method is taken from a real project. It contains the code of some particular functionality “as is”, and has been manually validated by the curators of BigCloneBench. Our approach combines the DeepClone model and information retrieval (IR) techniques to recommend real clone methods.

Recommending (real) code clones has various benefits. Code clones are useful for exploratory development, where the rapid development of a feature is required and the remedial unification of newly generated clones is not clearly justified (Kapser & Godfrey, 2008). Also, cloned code is expected to be more stable and poses less risk than new development. Hence, we believe that clone methods can be considered a useful component for neural code generation, as they can be used to capture the common coding practices of developers, which can be offered as code prediction and completion to the developer.

In this paper, we first elaborate our work on the DeepClone model with more details and perform extended evaluation. Then, we propose *Clone-Advisor*, which improves the re-usability of predicted clone method by recommending closely matching real clone methods for code completion tasks. We achieve this by using IR techniques to remove errors and noise in the predicted clone method. We believe that our approach can help in improving the quality of code prediction based on user input. In this work, we have made the following contributions:

1. We elaborate our previous work on DeepClone (Hammad et al., 2020a) model with more related work and details, and provide an evaluation of DeepClone output in terms of perplexity scores.
2. We refine the raw output of DeepClone model with a novel approach called Clone-Advisor, for recommending real clone methods, using an IR technique (TF-IDF) for retrieving the most relevant clone methods from a search corpus.
3. We quantitatively evaluate our refined approach in terms of accuracy and effectiveness by calculating various metrics. The overall results show that the refined approach, *Clone-Advisor* significantly improves the quality of the recommendations over the original ones from DeepClone.

## Related work

In this section, we present related work covering neural language modeling for code prediction, neural language generation techniques for code, machine learning approaches and recommendation systems in the field of code clones. Finally, we also discuss related literature in the field of syntax error detection and correction.

### Language modeling for code prediction

There exists no other technique in the literature, to the best of our knowledge, which models code clones for code prediction up to the complete method granularity. Language modeling has been explored for related tasks of token prediction, code suggestion, and code completion. Major examples include the work by White et al. (2015), where they applied Recurrent Neural Network (RNN) for Java source code modeling and prediction, and the approach by Boldt (2017) for modeling Java language method statements and English language datasets using LSTM. The latter compared the performance of the next token prediction task for code and English text, and found that method statements highly resemble and are comparable to English language sentences. While comparing natural language and source code, Hindle et al. (2016) discovered that software is much more repetitive and well structured than natural language. Hence, it is much simpler to model Java code by using n-grams rather than the English language. They compared the performance of language models on next element prediction task and demonstrated that n-gram models trained on Java dataset performed much better than n-gram models trained on English language dataset. Hussain et al. (2019) proposed a GRU-based model, which is used to perform code completion task till the whole line and generate source code suggestions. In our methodology, we develop a language model, which can predict the next set of tokens or complete clone method body (in contrast to the whole line) based on the code written so far. Hellendoorn & Devanbu (2017) noticed the poor performance of source code NLMs because of the large vocabulary size due to the high rate of new identifiers being defined, but argued that limiting vocabulary size is not a good strategy for source code NLMs. Instead, they proposed a nested scope, dynamically updatable, unlimited vocabulary count-based n-gram model, which outperforms the LSTM model on token prediction. In contrast, Karampatsis et al. (2020) solved the vocabulary size issue by applying byte-pair encoding (BPE) technique for modeling the code. They compared the performance of n-gram and Gated Recurrent Unit (GRU) language models on source code, and showed that GRU with BPE can outperform *n*-gram statistical models on code completion and bug detection tasks. Zhong, Yang & Sun (2019) applied LSTM with sparse point network for JavaScript code modeling and code prediction. Finally, Deep TabNine is a recently developed programming productivity tool, successfully fine-tuned by using GPT-2 on approximately two million GitHub files capturing numerous programming languages, to predict the next chunk of code.

### Neural language generation

Many techniques have been introduced to improve the quality of predicted text and code, though not specifically for clone method prediction. Hashimoto et al. (2018) proposed an approach to improve the predicted python code tokens, first by retrieving a training example based on the input (*e.g*., natural language description) and then editing it to the desired output (*e.g*., code). Code2vec (Alon et al., 2019) is a neural model representing snippets of code as code embeddings that helps in predicting method names based on method bodies. However, the purpose of our fine-tuned GPT-2 model is to predict next tokens/clone methods based on user input. Lancer (Zhou, Shen & Zhong, 2019) is a context-aware code-to-code recommendation tool leveraging a Library-Sensitive Language Model and a BERT model to recommend relevant code samples in real-time, by automatically analyzing the intention of the incomplete code. Lancer uses the BERT model to complete an incomplete code sample. It retrieves the relevant real code samples on the basis of Elastic search and rank them according to the deep semantic ranking scheme. The major difference with our methodology is that Lancer uses the BERT model, whose intention is to complete the missing tokens in incomplete code, while we are using the fine-tuned GPT2 model, which is used to predict next tokens based on the input. DeepClone is fine-tuned on both cloned and non-cloned code, while Lancer only uses clone methods for training. Moreover, we can predict a clone method from DeepClone model, whose functionality matches with the ground truth, even the context does not contain a method name, a scenario that is apparently not covered by Lancer.

### Machine learning approaches for clone detection and clone searching

Even though applying machine/deep learning-based approaches for clone prediction is a new idea, but these techniques have been extensively used previously for clone detection. However, the two techniques are not directly comparable. White et al. (2016) used a recursive neural network for clone detection. Wei & Li (2017) used the LSTM model for the functional clone detection problem by learning supervised deep features. CCLearner (Li et al., 2017b) extracts tokens from known method-level code clones and non-clones in a given codebase to train a classifier, which is then used to detect clones. Tufano et al. (2018) used a deep learning-based approach to automatically learn code similarities from different representations. Arammongkolvichai et al. (2019) proposed a method to increase the precision of code clone detection using machine learning techniques. They applied 19 clone class metrics to capture different characteristics of code clones and used them to train a decision tree model. DeepSim (Zhao & Huang, 2018) is a deep-learning approach, which measures similarity patterns between semantic matrices generated from functionally similar methods. Zhang et al. (2019) propose novel neural source code representation, which can capture the lexical, and syntactic knowledge of statements. This representation helps performing source code classification and clone detection. SourcererCC (Sajnani et al., 2016) is a token-based clone detector, and NiCAD (Cordy & Roy, 2011) is a text based hybrid clone detector. These clone detection tools can detect different types of clones of various granularity levels such as statements, method or file, in a given code corpus. However, in our case, we first generate and suggest clone methods by using the DeepClone model on the basis of user context. That method is a buggy snippet, which we feed to search for similar clone fragments. Therefore, we cannot empirically compare our methodology with clone detection approaches.

Kim et al. (2018) and Ragkhitwetsagul & Krinke (2019) propose effective code-to-code search approaches, which feed an original clone snippet and search for syntactically and semantically similar clone fragments in large code bases. In both approaches, BigCloneBench dataset is used to measure the accuracy. However, in our case, we feed a buggy snippet generated by DeepClone model, to search for similar clone fragments; therefore our results cannot be empirically compared with Kim et al. (2018) and Ragkhitwetsagul & Krinke (2019).

### Recommendation systems for code clones

Abid (2019) have previously used clone methods for code recommendation, however, the approach used a different similarity measure based on API calls for recommending relevant clone methods. Clones are generally considered to be harmful for a software system, and mainly researchers work on techniques for avoiding and eliminating clones (Yoshida et al., 2019; Wang & Godfrey, 2014; Basit et al., 2015; Basit, Hammad & Koschke, 2015; Hammad et al., 2020b). Clone refactoring recommendation systems have been developed for this purpose. For instance, Yoshida et al. (2019) proposed a proactive clone recommendation system for “Extract Method” refactoring, while Wang & Godfrey (2014) introduced an approach for automatically recommending clones for refactoring using a decision tree-based classifier.

### Syntax error detection and correction

Santos et al. (2018) present Sensibility, which leverages n-gram and LSTM language models to locate single token syntax errors and to suggest fixes for them. In our case, we propose a methodology to recommend correct fixes for the entire clone method. However, a perplexity metric is used here which is also common in our approach to measure the naturalness of the code. Wang et al. (2016) learn n-gram language models to detect uncommon usages of code. They do not provide a fix for those bugs. SEQUENCER (Chen et al., 2019) is a sequence-to-sequence deep learning model that aims at automatically fixing bugs by generating one line patches. Tufano et al. (2018) propose a deep learning model that aims at automatically fixing bugs by translating the entire buggy method into the corresponding fixed method. The maximum method length they considered is only 100 tokens. Lutellier et al. (2020) propose context aware neural machine translation (NMT) architecture to translate buggy code methods to correct code methods, which are partial code segments instead of full methods. In these approaches, models are trained to map an input sequence to an output sequence. So, the model is learned on a dataset, where every buggy snippet has its corresponding correct fix. In our case, language model is trained on IJaDataset, which contains millions of correct (*i.e.*, not buggy) source files. However, the model itself produces bugs while generating code because of its probabilistic nature. We measure TF-IDF scores by comparing the generated buggy clone method with real clone methods and recommend the most similar top-k methods. Because of all these differences, we cannot empirically compare our approach with any of the above approaches.

## Methodology

### Language modeling

In this section, we describe how we perform language modeling and how we construct the DeepClone model (Hammad et al., 2020a) by conducting a detailed empirical evaluation on the model quality and accuracy for the token prediction task.

#### Dataset preparation

For this work, we use a reduced version of IJaDataset containing only the source files whose clone method references exist in BigCloneBench (Svajlenko et al., 2014; Svajlenko & Roy, 2015; Svajlenko & Roy, 2016). BigCloneBench is the largest clone benchmark dataset, consisting of over 8 million manually validated clone method pairs in IJaDataset 2.0 (https://sites.google.com/site/asegsecold/projects/seclone)-a large Java repository of 2.3 million source files (365 MLOC) from 25,000 open-source projects. BigCloneBench contains references to clones with both syntactic and semantic similarities. It contains the references of starting and ending lines of method clones existing in the code repository. In forming this benchmark, methods that potentially implement a given common functionality were identified using pattern based heuristics. These methods were manually tagged as true or false positives of the target functionality by judges. All true positives of a functionality were grouped as a clone class, where a clone class of size *n* contains $\textstyle{{n(n - 1)} \over 2}$ clone pairs. The clone types and similarity of these clone pairs were later identified in a post-processing step. Currently, BigCloneBench contains clones corresponding to 43 distinct functionalities.

IJaDataset is a very large code base, and outside the scalability limits of most clone detection tools. However, the clone detection tools do not need to be executed for the entire IJaDataset, but only for the files containing reference clones in BigCloneBench. Svajlenko et al. (2014) (Svajlenko & Roy, 2015; Svajlenko & Roy, 2016) provide a reduced version of IJaDataset, only containing the relevant source files, and is distributed into a number of smaller subsets for clone detection. There is one subset per functionality in BigCloneBench. Each functionality’s subset includes all the files containing methods tagged as true or false positive of that functionality in the creation of BigCloneBench. Therefore each subset is a realistic subject system, containing both true and false positive clones.

**Figure 1:**
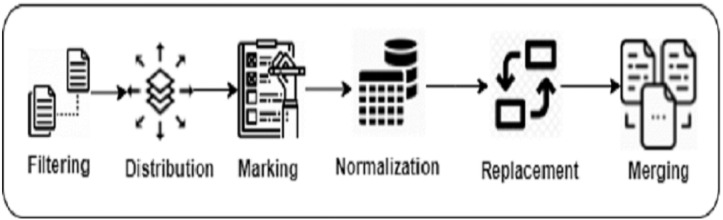
Data preparation steps of DeepClone methodology.

We performed pre-processing steps to build our mutually exclusive training, testing, and validation datasets. These steps took around 2 h on the whole dataset, which is quite negligible compared to other training processes. It has no overhead at run-time (when the recommendations are being generated). The training set is used to train DeepClone language model. After each training epoch, the trained model is evaluated on the validation set and its performance helps in assessing the convergence against hyper-parameters (*e.g.*, learning rate in gradient searches). The validation set is not used to learn any of the model’s parameters. The testing set is used for empirical evaluation of DeepClone model. Table S2 demonstrates the pre-processing steps on an example of binary search clone method. Similarly, Fig. 1 displays a pictorial representation of the data preparation steps.

**Filtering **We applied the following query to retrieve the list of source files containing true positive clone method references in IJaDataset, The functions table contains information about true and false positive clone methods, including filename, starting and ending line position of the clone method, the type id of the method. Whereas the clones table contains the list of true positive clone method pair information including syntactic similarity and validity measures.

select distinct a.functionality_id, b.type, b.name,

b.startline, b.endline from clones a

join functions b on a.function_id_one=b.id

union

select distinct a.functionality_id, b.type, b.name,

b.startline, b.endline from clones a

join functions b on a.function_id_two=b.id

**Distribution **We distribute the set of files into training, validation, and testing datasets using stratified sampling (Trost, 1986) to ensure that all types of clone methods appear in each dataset. We distribute the set of files existing in each functionality folder into three portions such that 80% goes to training, 10% to validation, and 10% to testing. Then, we copy those files into three separate folders of training, validation, and testing. If any of the file already exists in one of these folders, we keep only one copy to avoid file duplication in training and testing datasets.

Allamanis (2019) reported the negative impact of having the same file for both training and testing on model performance. Tables 1 and 2 depict the detailed statistics of our training, validation and testing datasets. We have only mentioned the titles of the functionalities in Table 1, and excluded further details such as functionality description, regular expressions used to obtain these methods, which can be obtained from the original sources (Svajlenko et al., 2014; Svajlenko & Roy, 2015; Svajlenko & Roy, 2016).

**Table 1:** Detailed statistics of datasets along with experimental results.

Id	Name	Files			Clone methods				Similarity	
		Training	Validation	Testing	Training	Validation	Testing	$\overline {PPL}$	*μ*	*σ* ^2^
2	Download from web	655	82	82	715	97	94	2.209	0.446	0.024
3	Secure hash	983	123	124	1,072	132	132	2.176	0.444	0.031
4	Copy file	2,088	260	261	2,454	306	295	2.267	0.372	0.031
5	Decompress zip archive.	4	1	0	8	1	0	–	0.392	0.043
6	Connect to FTP server	137	18	18	173	24	25	2.652	0.383	0.029
7	Bubble sort array	106	13	14	133	19	15	2.096	0.498	0.046
8	Setup SGV	19	2	3	19	2	3	4.362	0.458	0.045
9	Setup SGV event handler	6	1	2	7	2	2	3.085	0.310	0.040
10	Execute update and rollback.	349	44	44	567	56	71	2.278	0.415	0.030
11	Initialize Java eclipse project.	16	2	3	17	2	4	2.672	0.400	0.042
12	Get prime factors	16	2	2	17	2	2	3.923	0.586	0.044
13	Shuffle array in place	48	6	7	65	7	7	4.144	0.496	0.07
14	Binary search	251	31	32	315	54	34	2.814	0.537	0.017
15	Load custom font	19	2	3	21	2	3	2.982	0.414	0.029
17	Create encryption key files	14	2	2	17	2	2	2.931	0.378	0.04
18	Play sound	25	3	4	31	3	5	3.746	0.483	0.024
19	Take screenshot to File	69	9	8	82	12	9	3.049	0.421	0.030
20	Fibonacci	168	21	22	169	21	22	2.168	0.872	0.022
21	XMPP send message	18	2	3	20	2	3	3.147	0.484	0.024
22	Encrypt To file	49	7	8	59	8	8	2.406	0.420	0.028
23	Resize Array	224	27	29	317	44	36	2.484	0.487	0.031
24	Open URL in system browser	219	28	29	295	37	36	2.516	0.400	0.039
25	Open file in desktop application	54	9	7	82	12	8	2.517	0.376	0.037
26	GCD	16	2	3	18	2	3	6.686	0.597	0.030
27	Call method using reflection	294	37	37	329	39	41	2.183	0.402	0.041
28	Parse XML to DOM	122	15	16	157	21	19	2	0.435	0.031
29	Convert date string format	35	4	5	43	10	6	3.28	0.295	0.052
30	Zip files	783	97	99	1,119	136	135	2.272	0.411	0.027
31	File dialog	194	26	24	364	50	43	2.361	0.376	0.043
32	Send E-Mail	178	23	23	190	25	25	1.781	0.450	0.036
33	CRC32 File checksum	142	21	19	217	29	28	2.761	0.341	0.031
34	Execute external process	306	38	39	375	40	47	2.086	0.373	0.037
35	Instantiate using reflection	582	73	73	656	83	99	3.115	0.350	0.026
36	Connect to database	126	16	17	167	20	19	2.137	0.368	0.036
37	Load file into byte array	104	13	14	124	14	16	2.274	0.421	0.035
38	Get MAC address string	15	2	3	18	2	3	2.745	0.470	0.017
39	Delete folder and contents	175	23	24	218	27	30	3.022	0.497	0.032
40	Parse CSV file	125	14	18	161	16	25	2.004	0.433	0.038
41	Transpose a Matrix.	333	42	41	395	45	50	2.527	0.388	0.051
42	Extract matches using Regex	337	43	44	405	47	48	2.921	0.420	0.028
43	Copy directory	65	8	10	118	15	15	2.289	0.480	0.024
44	Test palindrome	15	1	3	133	16	19	1.668	0.903	0.04
45	Write PDF file	122	15	15	129	15	15	2.304	0.431	0.034

**Table 2:** Final distribution of BigCloneBench dataset.

	Files	Clone methods	Tokens
Training	9,606	11,991	16,933,894
Validation	1,208	1,499	2,130,360
Testing	1,234	1,502	2,235,982
Total	12,048	14,992	21,300,236

**Marking **Researchers in the past have used meta-tokens to mark special sections of data. Pichotta & Mooney (2016) placed $\langle$S $\rangle$ and $\langle$/S $\rangle$ meta-tokens in modeling sentences for prediction. Chen et al. (2019) inserted $\langle$START_BUG $\rangle$ and $\langle$END_BUG $\rangle$ meta-tokens in the buggy lines of the source code to help in automatic program repair. We have also marked the regions of the true positive clone methods by placing the meta-token $\langle$soc $\rangle$ at the start, and $\langle$eoc $\rangle$ at the end of a clone method in the IJaDataset files, by tracing the clone method references from the BigCloneBench dataset.

**Normalization **We have adapted the Javalang (https://github.com/c2nes/javalang) Python library, which contains a lexer and parser for the Java 8 programming language, to normalize the input source code by removing whitespaces, extra lines, comments, as well as to tokenize the code.

**Replacement **For each set of files, we have replaced integer, float, binary, and hexadecimal constant values with the $\langle$num_val $\rangle$ meta-tokens. Similarly, we replace string and character values with $\langle$str_val $\rangle$. This reduces our vocabulary size, leading to faster training of the model. This is a common technique for data preparation (White et al., 2015; Dam, Tran & Pham, 2016; Karampatsis et al., 2020).

**Merging **We merge all the tokenized data existing in the training, validation and testing folders, and place them into three text files: train.txt, valid.txt and test.txt. These files are called as Experimental Datasets. These tokens are separated by the space character. Table 2 provides the relevant statistics of the experimental dataset.

#### Neural language models for code clones

A number of techniques are available for developing a LM for BigCloneBench dataset such as *n*-gram statistical model (Hellendoorn & Devanbu, 2017), LSTM (Hochreiter & Schmidhuber, 1997), GRU (Cho et al., 2014), GPT-2 (Radford et al., 2019b); as well as parameter settings for training those models. We could not evaluate all the possible combinations (hundreds) and especially very large scale models/training due to the resource limitations. We selected GRU (Cho et al., 2014) and GPT-2 (Radford et al., 2019b) as they have been reported to outperform other comparable models with recommended configurations. In the following sections we describe the two models.

**Gated Recurrent Units (GRU) **Gated recurrent units (GRUs) are a gating mechanism in RNNs (Cho et al., 2014), which is similar to LSTM (Hochreiter & Schmidhuber, 1997) but has a forget gate and fewer parameters as it lacks an output gate. However, it is known to perform better than LSTM on certain tasks. To prepare our dataset (“Dataset preparation”), we applied the recently proposed configuration settings for GRU deep learning model by Karampatsis et al. (2020), which outperforms n-gram models on code completion and bug detection tasks.

Byte-pair encoding (BPE) technique is generally used to solve the unlimited vocabulary problem (Allamanis & Sutton, 2013). This problem makes it infeasible to train LMs on large corpora. Researchers (Hindle et al., 2016; White et al., 2015) have applied several other techniques such as replacing low frequency tokens, and replacing all code tokens not appearing in training data set to reduce the vocabulary size and to build an open vocabulary at the time of testing, with unknown tokens. This approach is not practical for source code where software developers continuously introduce new variables, objects and function names. These traditional language models in NLP mostly operate at the token level (Dam, Tran & Pham, 2016; Sundermeyer, Ney & Schlüter, 2015), predicting one token at a time. But for code, this strategy leads to large vocabulary sizes, because identifiers in programming languages often correspond to entire phrases in natural language. Because the number of unique identifiers increases with the size of the corpus (Allamanis & Sutton, 2013), this problem makes it infeasible to train code LMs on large corpora.

In order to solve these issues, many NLP models have used linguistically-motivated subwords (Bazzi, 2002, Creutz et al., 2007; Luong, Socher & Manning, 2013; Mikolov et al., 2012). Sennrich, Haddow & Birch (2015) first adapted the algorithm for word segmentation, so that instead of merging pairs of bytes, it merges pairs of characters or character sequences. The learnt segmentation was used in their neural translation system and resulted in improved translation of rare words. Mikolov et al. (2012) found that subword models improved upon character models. Sennrich, Haddow & Birch (2015) adapted BPE to decompose words into subwords, improving rare word translation. The vocabulary of subword units is learnt before training the NLM by segmenting a corpus of code. This is done in such a way that more frequent character *n*-grams are more likely to be included in the vocabulary of subword units. This strategy results in a core vocabulary of subword units that occurs frequently in the corpus and captures statistical patterns of characters within identifiers. Subword segmentation *via* BPE (Babii, Janes & Robbes, 2019; Karampatsis et al., 2020) outperforms traditional approaches (Dam, Tran & Pham, 2016; Sundermeyer, Ney & Schlüter, 2015) operating at token level, *n*-gram, cache model and so on, for both small and large datasets.

BPE algorithm was originally designed for data compression, in which bytes that are not used in the data replace the most frequently occurring byte pairs or sequences (Gage, 1994). BPE starts by splitting all the words in characters. The initial vocabulary contains all the characters in the data set and a special end-of word symbol @@, and the corpus is split into characters plus @@. Then, it finds the most common pair of successive items in the corpus (initially characters, then tokens). This pair is merged in a new token which is added to the vocabulary; all occurrences of the pair are replaced with the new token. The process is repeated n times, which is called a merge operation (MO).

We applied static settings with a large training set (50 epochs, 64 mini-batch size) and chose 10,000 BPE MOs as it performs better than other BPE MOs such as 2,000 and 5,000. Static settings have been used to train a model on a fixed training corpus, and later evaluated on a separate test dataset. To train the LM, we first learn encoding by using the training set with the help of subword library (https://github.com/rsennrich/subword-nmt). Then, we segment the training, validation, and test sets using the learnt encoding, and apply the MOs from BPE to merge the characters into subword units in the vocabulary.

**Generative Pretrained Transformer 2 (GPT-2) **OpenAI developed a large-scale unsupervised LM called GPT-2 (Generative Pretrained Transformer 2) (Radford et al., 2019a; Radford et al., 2018; Radford et al., 2019b) to predict several sound sentences of realistic text by extending any given seed. It is a direct scale-up of GPT, with more than ten times the parameters and training data. We focus on fine-tuning a GPT-2 transformer (Radford et al., 2019b) pre-trained model for predicting code tokens, even though it has been trained on English language. Fine-tuning works well, if a pretrained model has been trained over a large corpus, and there is an overlap of vocabulary between languages (see, for instance, Wu & Dredze, 2019; Lample & Conneau, 2019; Ruder, Vulic′ & Søgaard, 2019). GPT-2 is effective to fine-tune on Java programming language, as it employs byte pair encoding (BPE) to construct its vocabulary. So, all tokens in Java language can be mapped to the vocabulary set. We applied fine-tuning of a pre-trained model on IJaDataset as there exists a large amount of overlapping vocabulary with the English language.

GPT-2 has demonstrated impressive effectiveness of pre-trained LMs on various tasks including high quality text generation, question answering, reading comprehension, summarization, and translation (Radford et al., 2019b). It was also noticed that, in general, better pre-trained models lead to better performance on fine-tuned or transfer tasks (Peters, Ruder & Smith, 2019). Fine-tuning is one approach to transfer learning, which is to adjust feature weights according to the new dataset on some already trained model. Previously, GPT-2 has been successfully fine-tuned on different types of datasets. Shrestha & Csallner (2021) have fine-tuned the pretrained GPT-2 model on Simulink model files to generate Simulink models. Kim et al. (2020) have used GPT-2 for code prediction by revealing the syntactic structure of code to the network. Ziegler et al. (2019) have applied a reinforcement learning method on the 774M GPT-2 model to support human-preferred text more often. Lee & Hsiang (2019) fine tuned 345 M, a GPT-2 based pre-trained model of medium version, to patent claim generation by providing various experimental results for qualitative analysis and future research. Deep TabNine, a software programming productivity tool to predict the next chunk of code, has been successfully fine-tuned by using GPT-2 on approximately two million GitHub files capturing numerous programming languages. DeepClone is initially inspired by Deep TabNine, and we have fine-tuned for only those files of IJaDataset, which contains true positive clone methods from BigCloneBench dataset.

GPT-2 also has built in BPE tokenizer. We selected a small version of GPT2 (GPT2-117) as our base model, as it does not take too much time and resources to fine-tune, and is enough to evaluate our approach. The GPT2-117(Radford et al., 2019b) pre-trained model has vocabulary size of 50,257, 117 M parameters, 12-hidden layers, 768-hidden states, and 12-attention heads. We have fine-tuned our GPT-2 based model on the partition of a GPU-1080Ti cluster (276 CPU cores, 329,728 CUDA cores, 5.9 TB memory) (https://userinfo.surfsara.nl/) for approximately 9 h by using HuggingFace Transformer Library. In our experiment, we have performed training and evaluation with batch size per GPU of one for five epochs. We have used a learning rate of 5e − 5 and the gradient accumulation steps (number of update steps to accumulate before performing a backward/update pass) as 5. Default values have been used for other hyper-parameters, as mentioned in the language modeling code (https://github.com/huggingface/transformers/tree/master/examples/pytorch/language-modeling).

#### Comparative evaluation: GRU vs GPT-2 based models

We have performed both intrinsic and extrinsic evaluations of GRU and GPT-2 based models to compare their performance. In order to measure the quality of the models (*i.e.*, intrinsic evaluation), we have calculated the perplexity scores (as done in related work (Zaremba, Sutskever & Vinyals, 2014; White et al., 2015)), which is an inverse of cross-entropy (as used in (Hellendoorn & Devanbu, 2017; Karampatsis et al., 2020)). Perplexity is a measurement of how well a given LM predicts sample data. It estimates the average number of code tokens to select from at each point in a sequence (Allamanis & Sutton, 2013). It is a natural evaluation metric for LMs, which represent a probability distribution over a subsequence or an entire dataset (Eq. (1)):

(1) $$P(L) = exp\left ( - \displaystyle{1 \over M}\sum\limits_i^M \log P({t_i}|{t_0}:{t_{i - 1}})\right)$$

*P*(*t*_*i*_ | *t*_0_:*t*_*i*__− 1_) is the conditional probability assigned by the model to the token *t* at index *i*. By applying *log* of conditional probability, cross-entropy loss is calculated. *M* refers to the length of tokens. Hence, perplexity is an exponentiation of the average cross entropy loss from each token [0, *M*]. We calculate the perplexity on the validation set (**P1**) and the testing set (**P2**) for GRU and GPT-2 based models, which clearly displays that the GPT-2 based model outperforms the other by a large margin (Table 3).

**Table 3:** Comparative evaluation results for GPT-2 and GRU models.

	Perplexities			Accuracies			
Model	Validation (P1)	Test (P2)	MRR (%)	Top 1 (%)	Top 3 (%)	Top 5 (%)	Top 10 (%)
GPT-2	2.145	2.146	84.329	77.808	90.040	92.766	94.999
GRU	13.92	13.86	73.507	66.948	79.0715	82.02	84.787

We have further measured the performance of both models on specific tasks such as token prediction (*i.e.*, extrinsic evaluation). Given a number of code sequences as input, we have collected the top ten predictions from GRU and GPT-2 based models, and computed the top-k accuracy (the fraction of times the correct prediction appears in the top k predictions) for k ∈ [1, 10]. Moreover, we have measured the Mean Reciprocal Rank (MRR) scores of both language models (LM), which has been used by many researchers (Karampatsis et al., 2020, Hellendoorn & Devanbu, 2017) for evaluating code prediction. For each prediction done by the LM, we have collected a ranked list of ten predictions. For each of those lists, the reciprocal rank corresponds to the multiplicative inverse of the rank of the first correct answer. MRR in turn is the average of reciprocal ranks for all the input sequences used in the evaluation.

Table 3 shows the top-k accuracies as well as the MRR scores. Clearly, the results suggest that the GPT-2 based model performs more accurately compared to the GRU based model on pre-processed Java source code containing clone methods. The table also indicates that there is almost 78% chance to get a correct token in the first option, and 95% chance to have a correct output in the top-ten predicted outcomes for GPT-2 based model. To further quantify the accuracy of our models for token prediction task, we report an MRR score of 83%, which indicates an excellent performance in evaluating a ranked list of predictions for GPT-2 based model. As GPT-2 based model gives us highest performance in terms of perplexity on the validation set (**P1**) and test set (**P2**), MRR, and top-k accuracy, we selected this model for our approach and named it DeepClone model.

DeepClone based on GPT-2 produces more accurate results on the token prediction task than GRU.

### Recommending real clone methods

In this section, we describe how we predict clone methods (*i.e.*, the DeepClone output) based on user input and how we recommend real clone methods based on the resulting DeepClone output.

#### Clone prediction

For predicting a clone method based on user input, there exist several text generation methods such as beam search (Vijayakumar et al., 2018), sampling with temperature (Ackley, Hinton & Sejnowski, 1985; Ficler & Goldberg, 2017), top-k sampling (Fan, Lewis & Dauphin, 2018) and nucleus sampling (Holtzman et al., 2019). All these methods have a specific decoding strategy to shape the probability distribution of LM with higher probabilities assigned to higher quality texts. We select nucleus sampling as it is claimed to be best the strategy for predicting large amount of high quality text, comparable to human written text (Holtzman et al., 2019). By using a fine-tuned model and nucleus sampling, we can expect a coherent set of code tokens for clone method prediction. Holtzman et al. (2019) have also achieved coherent text generation results with similar settings. We have mentioned sample DeepClone output from our results in Tables S1, S3, S4.

#### Clone recommendation

DeepClone is the first step for code prediction that raises the granularity level to complete clone methods. However, with the probabilistic model alone, we cannot expect exactly the same clone method being predicted or completed as the one used in training. In prediction tasks, generating well-formed outputs is challenging, which is a well-known problem in natural language generation (Hashimoto et al., 2018). However, the desired output might be a variation of another, previously observed sample (Hashimoto et al., 2018).

**Figure 2:**
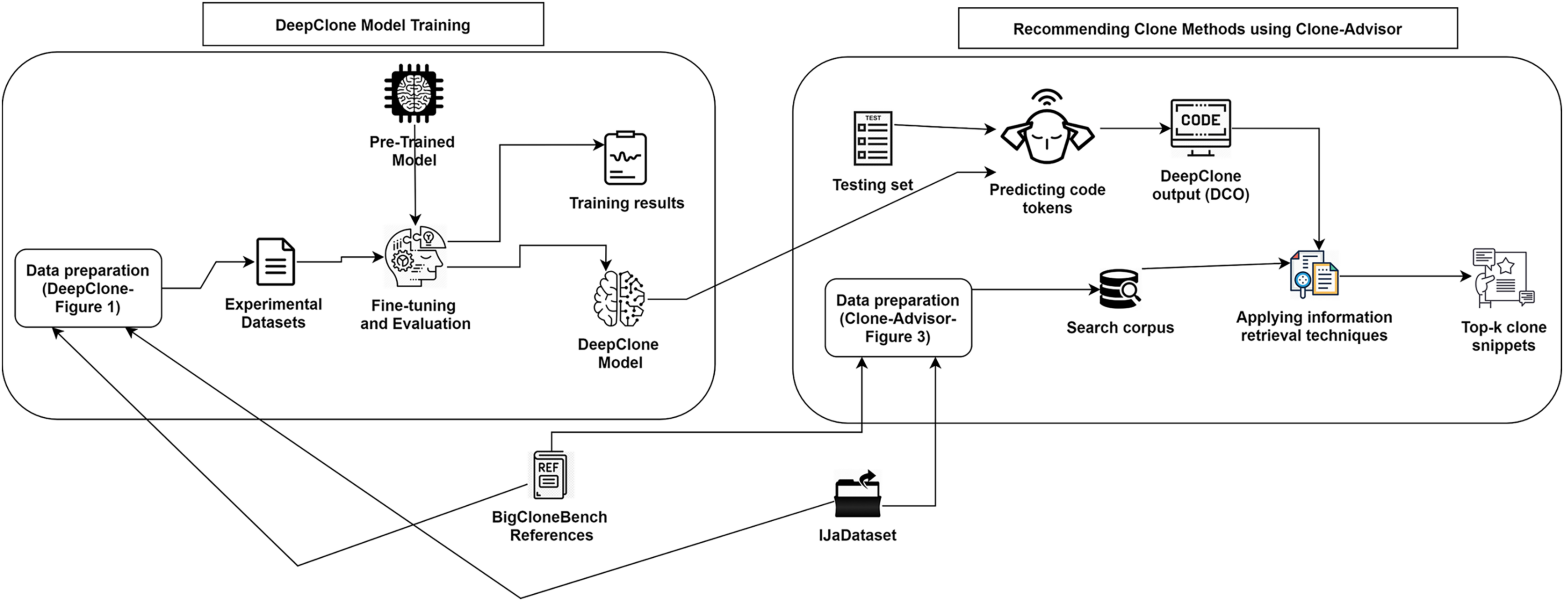
DeepClone training process and methodology of Clone-Advisor for recommending real clone methods.

We propose a methodology called Clone-Advisor, for recommending real clone methods based on given code context by applying an IR technique over the previously described DeepClone output. The IR technique retrieves real clone methods from the search corpus, which are most similar to the initially predicted clone method from DeepClone model. Figure 2 displays a pictorial representation of our methodology to predict real clone methods. In their raw form, we obtain DeepClone output, ground truth, and top-ten samples from the current methodology in an unformatted style, where code tokens of the clone method are separated only by space characters. To make this output readable, we have formatted the code by using an online tool (https://www.tutorialspoint.com/online_java_formatter.htm) along with little manual editing (which we plan to automate in future). We have mentioned sample examples from our results in Tables S1, S3, S4. We describe the details of our methodology in the following subsections.

**Figure 3:**
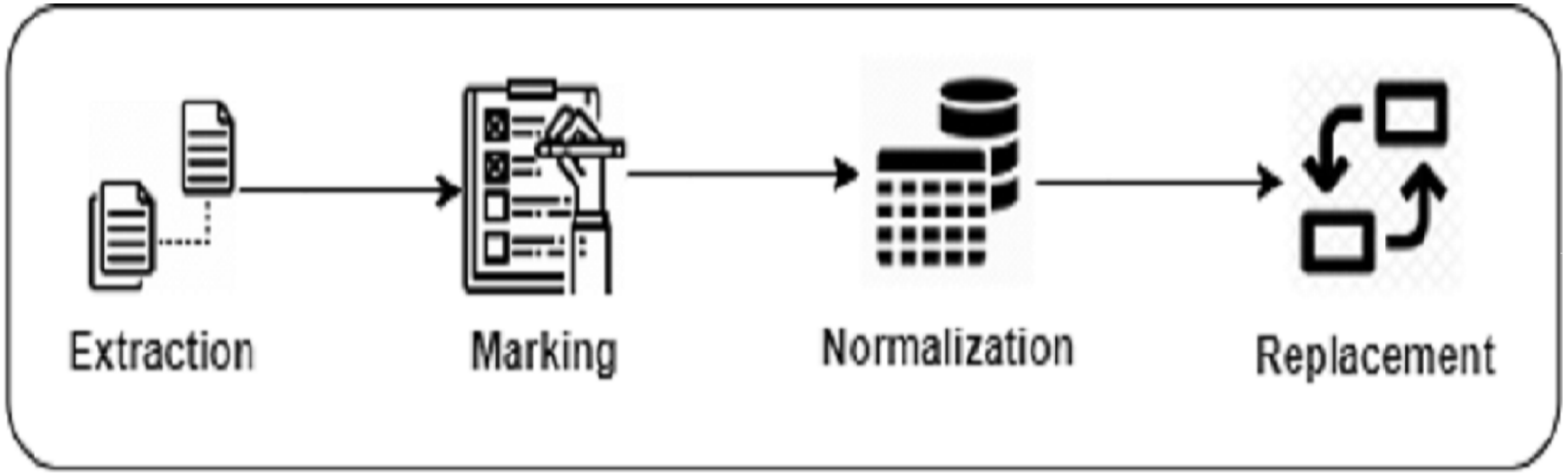
Data preparation steps of Clone-Advisor methodology.

**Building the Search Corpus **We build our search corpus from BigCloneBench and IJaDataset (Svajlenko et al., 2014; Svajlenko & Roy, 2016), which we also previously used to train DeepClone. We perform several pre-processing steps to build our search corpus, which is similar to what we have followed in “Language modeling”. First, we extract the details of a total of 14,922 true positive clone methods (*Extraction*). Next, we trace them in IJaDataset files, by following their references from the BigCloneBench dataset, and put them in our search corpus list by placing meta tokens $\langle$soc $\rangle$ at the start, and $\langle$eoc $\rangle$ at the end of each clone method (*Marking*). These meta tokens are also part of the DeepClone output, so inserting them in the search corpus clone method list helps in making a fair comparison. Afterwards, we normalize each clone method code by removing whitespaces, extra lines, comments, as well as tokenizing (*Normalization*) by adapting the Javalang (https://github.com/c2nes/javalang) Python library, which contains a lexer and parser for the Java 8 programming language. Finally, for each clone method, we replace integer, float, binary, and hexadecimal constant values with the $\langle$num_val $\rangle$ meta-token (*Replacement*). Similarly, we replace string and character values with $\langle$str_val $\rangle$. Again, this is just to ensure to have fair comparison, as DeepClone output is also in this normalized format. Figure 3 displays a pictorial representation of building a search corpus.

**Retrieving Clones from the Search Corpus **The output from “Experimental design” contains the set of tokens from the user input, along with the predicted tokens up to the $\langle$eoc $\rangle$ token. In this step, we extract only those tokens, which are between $\langle$soc $\rangle$ and $\langle$eoc $\rangle$ tokens (inclusive) from the DeepClone output (see the DeepClone output steps in Tables S1, S3 and S4). We apply an IR technique to retrieve top-ten results from the search corpus matching the clone method predicted from DeepClone model. IR techniques, in general, are used to discover the significant documents in a large collection of documents, which match a user’s query. Their main goal is to identify the significant information that satisfies the user information needs. An IR-based code retrieval method in particular usually extracts from a query a set of keywords and then search for the keywords in code repositories (Nie et al., 2016).

The selected IR technique is based on TF-IDF word embeddings for retrieving the real clone methods most similar to the DeepClone output. TF-IDF (Term Frequency-Inverse Document Frequency (Dillon, 1983)) is a technique often used in IR and text mining. A survey conducted in 2015 showed that 70% of text-based recommendation systems in digital libraries use TF-IDF (Beel et al., 2016). Similarly, in the past many researchers have applied TF-IDF to retrieve code elements (Kim et al., 2018; Luan et al., 2019). TF-IDF is a weighting scheme that assigns each term in a document a weight based on its term frequency and inverse document frequency. In our context, TF-IDF is looking at the term overlap, *i.e.*, the number of shared tokens between the two clone methods in question (and also how important/significant those tokens are in the clone methods). We use TF-IDF with unigrams as terms to transform clone methods into numeric vectors, that can easily be compared by quickly calculating cosine similarities. If a term appears frequently in a clone method, that term is probably important in that method: term frequency is simply the number of times that a term appears in a method. However, if a term appears frequently in many clone methods, that term is probably less important generally. Inverse-document frequency is the logarithmically-scaled fraction of clone methods in the corpus in which the term appears. The terms with higher weight scores (high TF *and* IDF) are considered to be more important. We first transform clone methods existing in the search corpus and the DeepClone output into TF-IDF vectors using Eq. (2).

(2) $$TF - IDF(i,j) = (1 + \log (TF(i,j)).\log \left(\displaystyle{J \over {DF(i)}}\right)$$

where *TF (i, j)* is the count of occurrences of feature *i* in clone method *j*, and *DF (i)* is the number of clone methods in which feature *i* exists. *J* is the total number of clone methods. During retrieval, we create a normalized TF-IDF sparse vector from the DeepClone output as query, and then take its dot product with the feature matrix. Since all vectors are normalized, the result yields the cosine similarity between the feature vectors of the query and of every clone method. We then return the list of clone methods ranked by their cosine similarities.

## Empirical evaluation

In this section we describe the evaluation of DeepClone on additional aspects of the model. Furthermore, we empirically evaluate our approach of recommending real clone methods by presenting experimental design, various research questions and experimental results.

### DeepClone model

**Figure 4:**
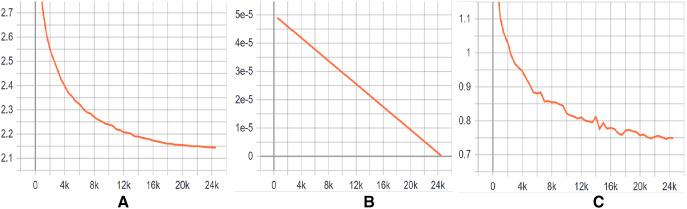
(A–C) Training graphs.

**Training Evaluation **We have evaluated the training phase as a indication of how well the optimization performed in our case. We have measured the performance of DeepClone model at each checkpoint, *i.e.*, per 500 logging steps, in terms of perplexity on the validation set. The decreasing and stabilizing trend on perplexity can be seen in Fig. 4A, with the lowest perplexity **P1** (2.145) at step 24,500. Supporting this, there is a clear convergence to zero in the learning rate after each checkpoint, as depicted in Fig. 4B. Learning rate is a useful indicator for determining how quickly a neural network model learns a problem by adjusting the weights of the network according to the value loss function. Loss function, in turn, calculates the model error. This measure identifies how well a model predicts the expected outcome for any data point in the training set. GPT-2 particularly uses the cross-entropy loss function as a probability value between 0 and 1. Figure 4C displays a convergence of training losses after each checkpoint, which indicates how well the model behaves after each checkpoint of optimization. At step 24,500, the loss value is finally minimized to 0.75, implying a well trained model. The training steps for the fine-tuning of our GPT-2 based model are shown in Fig. 2. In summary, all the measurements indicate a successful training process and an accurate model. We have published our training results online (https://tensorboard.dev/experiment/tk1XqDi8RMqtrMjmVyQ9Sg).

The DeepClone model has been successfully fine-tuned on the BigCloneBench dataset by using a powerful GPT-2 based pre-trained model.

**The Effect of Using Clone Markers **In this section we discuss the perplexity of our model on the testing dataset. However, in contrast to the our perplexity measurement in the previous sections, this time we have excluded the clone method markers (*i.e.*, $\langle$soc $\rangle$ and $\langle$eoc $\rangle$) from our data. Our motivation for this additional measurement is as follows. Hindle et al. (2016) observed predictable statistical properties in the source code due to its repetitive nature, and proposed n-gram language models to capture and leverage these for software engineering tasks. A good model should be able capture the patterns in the dataset very well, which is particularly important for the task of clone method prediction. In Table 3, we observe a 3.6% increase in perplexity when comparing the original measurement of 2.146 (**P2** and the one without clone markers of 2.182 (**P3**) see Table 4). This means that our model performs better with explicitly marked clone methods.

The prediction capability of DeepClone is better on code which has marked clone methods.

**Table 4:** Perplexities measured while evaluating the DeepClone model.

P3	P4	P5	P6
2.182	2.410	2.767	2.996

**Evaluation per Clone Method **We have calculated the average perplexity ( $\overline {PPL}$) for each functionality type (see Table 1) to assess which clone method snippets are more predictable than others. We have first extracted the code snippet for each type of clone method in the testing dataset, and averaged the perplexity score per funcionality type as an indicator of prediction likelihood (Table 1). We have also analyzed several factors which can potentially affect the perplexity scores. BigCloneBench contains a syntactic similarity score for each clone method pair, which is calculated using a line-based metric after normalization. We have calculated the mean (*μ*) and variance (*σ*^2^) values to determine the overall syntactic similarity of all the clone methods per functionality type in Table 1.

We observe that the perplexity scores vary according to the syntactic similarity between clone methods, as well as the number of clone method snippets in the training set. From the results, we can see, for instance, the “Test palindrome” (*i.e.*, checking whether a string is a palindrome) methods (number 44) have the lowest perplexity score. They can therefore be well predicted by DeepClone. We attribute this to the high mean syntactic similarity (0.903 *±* 0.040) among those types of clone methods, and the relatively small number of snippets (133) used in training. Too few snippets in the training may lead to (a) high perplexities and low predictability *e.g.*, for “GCD” (greatest common denominator, number 26) and (b) no evaluation performed for “Decompress zip archive” (number 5). We however believe other factors can also can also affect the perplexity score. In BigCloneBench, there are many false positive clone methods and other non-clone code snippets, which may be syntactically similar to true positive clone methods. Other factors such as clone types and hyper-parameters for GPT-2 are left to be explored in future work.

For the majority of the clone methods, DeepClone achieves a successful prediction.

**Non-Clone Methods *vs* Clone Methods **Allamanis (2019) noticed the negative correlation between perplexity and code duplication in the data for language modeling. In order to quantify this in our case, we have calculated the perplexity scores for all the clone method snippets and non-clone method snippets in the testing dataset. We have extracted clone method snippets by tracing the tokens between the clone markers. The remaining part of the dataset was considered to be a part of non-cloned code. We have then calculated the perplexity for each snippet. Finally, we have averaged the perplexities for both types of code snippets. In Table 4, **P4** represents the average perplexity score for the clone method snippets, and **P5** for the non-cloned method snippets. We have performed one-tailed Wilcoxon rank sum test to statistically compare **P4** with **P5**, which indicates that P4 is indeed less than P5 (*p*¡ 0.001). We conclude that DeepClone correctly predicts clone method snippets much better than non-cloned snippets in general.

DeepClone predicts clone code method snippets more accurately than non-clone ones.

**Performance on Other Datasets **We have evaluated the performance of DeepClone on another Java dataset to assess its applicability and generalizability. We have used Allamanis & Sutton (2013) corpus that contains over 14 thousand popular Java projects from GitHub. As a baseline, we have focused only on 38 test projects that have been used in previous studies (Hellendoorn & Devanbu, 2017; Karampatsis et al., 2020). We have followed the same steps for dataset preparation as mentioned in “Dataset preparation”, *i.e*., normalization, replacement, and merging. Note that this dataset does not contain clone markers as no corresponding clone reference benchmark is available. As the main purpose of clone markers is to help in predicting clone methods, they will not severely affect the results of predicting next tokens, also remarked in “DeepClone model”. On this dataset, we have achieved a perplexity of 2.996 (**P6**), equivalent to a cross-entropy of 1.097. We have further calculated additional accuracy measures: MRR (81.523%), top-1 (74.416%), top-3 (87.613%), top-5 (90.704%), and top-ten (93.152%). These results (see Table 5) outperform the static settings of previous studies (Hellendoorn & Devanbu, 2017, Karampatsis et al., 2020). This indicates that DeepClone model is very successfully fine-tuned with GPT-2 over Java corpus in general, and it contains an excessive amount of overlapping vocabulary with the selected corpus. We consider this as a good indicator for the generalizability of our approach on any Java code.

DeepClone is fine-tuned well with GPT-2 over potentially any general Java *corpus*.

**Table 5:** Performance of Allamanis & Sutton (2013) dataset on different models.

Model	Settings	Cross-Entropy	MRR (%)
LSTM/300 (Hellendoorn & Devanbu, 2017)	Static	3.22	66.1
LSTM/650 (Hellendoorn & Devanbu, 2017)	Static	3.03	67.9
BPE NLM (512) (Karampatsis et al., 2020)	BPE 10,000, Static, Small train	4.77	63.75
DeepClone	“Language modeling”	1.097	81.523

### Recommending real clone methods

We have performed an extensive quantitative evaluation of DeepClone output and Clone-Advisor recommendations with respect to various aspects: naturalness of the output, accuracy for finding exact matches, accuracy for finding the first correct functionality match, and accuracy for finding multiple correct functionality matches. We measure perplexity to identify the naturalness of the code. There are several metrics which are used to evaluate the performance of information retrieval systems such as precision, MRR, top-k accuracy, NDCG and recall. In this paper, we focus on the problem of how many relevant results are retrieved in the top-k retrieved clone methods. We emphasize on the quality of the retrieved answers in the top-k results. An ideal information retrieval system should hit more of the relevant records and place them at the top of the results. If a developer finds a relevant implementation of some functionality in the top-k retrieved results, it will be enough for him to move forward. Therefore, performance measures such as recall are not of major concern, as they are used to identify whether information retrieval systems miss in reporting some relevant result or not. Previously, there were also many researchers, who did not report recall because of the similar nature of the problem (Keivanloo, Rilling & Zou, 2014; Lv et al., 2015; Gu, Zhang & Kim, 2018; Yan et al., 2020). Based on these reasons, we choose MRR, top-k accuracy and precision metrics to determine the performance of our information retrieval system.

#### Experimental design

We have performed a small scale (100 context queries) experiment to predict next token subsequences by choosing different subsequence sizes such as 10, 20, 30, 50, and 100 tokens. Among these, subsequences with size 20 gave us best results in terms of top-k accuracy and MRR. We have extracted subsequences of 20 tokens from the testing dataset, and moved the sliding window one step ahead to obtain further subsequences. From these we have selected 735 subsequences containing a total of 14,700 tokens, in which soc token is a part of each subsequence, which indicates a start of clone method. We have passed these subsequences one by one to DeepClone model, and kept on predicting new tokens with nucleus sampling (threshold value 0.95) until the meta-token eoc (*i.e.*, end of clone) appeared. We have used the text generation script (https://github.com/huggingface/transformers/blob/master/examples/pytorch/text-generation/run_generation.py) of HuggingFace Transformer Library in this case. Note that certain parameters, such as the number of subsequences and size of tokens in each subsequence are chosen to perform a preliminary evaluation, which can be fine-tuned and optimized in a follow-up study. The main aim is to demonstrate the feasibility of our methodology for predicting and recommending clone methods.

#### Research questions

The objective of the empirical evaluation is to investigate the overall effectiveness of our approach in terms of DeepClone output and Clone-Advisor recommendations. We also want to investigate the benefit of distinctive features of our approach. We have identified the following research questions:

**RQ1: Which of the output, DeepClone output or Clone-Advisor recommendations, are considered to be more “natural”? **The objective of this RQ is to measure and analyze perplexity scores for the DeepClone output *versus* the Clone-Advisor recommendations around these angles of naturalness and potential bug density. In previous work, it has been observed that n-gram language models can detect defects as they are less “natural” than correct code (Ray et al., 2016). Similarly, Karampatsis et al. (2020) have noted that defective lines of code have a higher cross-entropy (~perplexity, to be explained later in this section) than their correct (ed) counterparts. By considering it, we expect the DeepClone output to have a relatively higher perplexity, because it is considered to be a buggy snippet as generated from probabilistic language models.

**RQ2: To what extent do Clone-Advisor recommendations exactly match with the ground truth? **The main purpose of this RQ is to inspect top-ten Clone-Advisor recommendations from Clone-Advisor and identify how much of them exactly match with the ground truth. For this purpose, we collect the top ten Clone-Advisor recommendations retrieved by Clone-Advisor, and compute the top-k accuracy (the fraction of times the ground truth clone method appears in the top k Clone-Advisor recommendations) for k ∈ [1, 10]. Moreover, we measure the Mean Reciprocal Rank (MRR) scores for the recommendations. A simplified description of MRR is that it averages top-k accuracy across various k. In this specific scenario k ∈ [1, 10] since the methodology output a list of top-ten Clone-Advisor recommendations. The MRR is a rank-based evaluation metric, which produces a value between 0 and 1, where a value closer to 1 indicates a better clone method recommendation system. The reciprocal rank of a query response is the multiplicative inverse of the rank of the first correct answer, while MRR is the average of reciprocal ranks. We have further calculated top-k accuracy and MRR involving an exact match of the Clone-Advisor recommendations with the ground truth.

**RQ3: Do the Clone-Advisor recommendations’ functionalities match those of the ground truth?**

Another distinctive feature of our approach is that the Clone-Advisor recommendations’ functionality is matched with the ground truth. In order words, they are the clones of the ground truth. BigCloneBench contains references of multiple implementations (*i.e.*, clones) of specific functionalities. It contains validated clone methods belonging to 43 different functionalities, for instance, “copy file” functionality contains 3,055 different implementations. Further details can be found from our previous paper (Hammad et al., 2020a), and BigCloneBench dataset. Hence, it is possible to have Clone-Advisor recommendations that do not exactly match the ground truth but match its functionality. For instance, Table S1 displays top-1 and top-2 clone methods belonging to the same functionality as the ground truth (GT). So, both implementations can potentially satisfy the user’s need. For this purpose, we extract the functionality id of the ground truth and recommended list of top-k clone methods against each context. We calculate top-k accuracy and MRR accordingly.

**RQ4: What percentage of the Clone-Advisor recommendations’ functionalities match with the ground truth? **The main aim of this RQ is to identify more than one correct result in the top-k Clone-Advisor recommendations. Oftentimes developers need to analyze multiple correct results from the search list. For this purpose, we compute the precision, which is the percentage of relevant results in the top-k Clone-Advisor recommendations for each query:

(3) $$Precision@k = \displaystyle{1 \over {|Q|}}\sum\limits_{|Q|}^{i = 1} \displaystyle{{|relevan{t_{i,k}}|} \over k}$$

where *relevant*_*i*,*k*_ represents the relevant search results for query i in the top k returned results, and Q is a set of queries. Precision shows the relevance of the Clone-Advisor recommendations to the queries with respect to the ground-truth functionalities; the higher the value, the more relevant the results are.

**RQ5: What is the performance of Clone-Advisor on code samples, which do not exist in BigCloneBench dataset? **The objective of this RQ is to investigate the overall effectiveness of our approach on other code samples, with the prerequisite that they belong to the same functionality group present in the BigCloneBench dataset (*i.e.*, the dataset we train our system on). For this purpose, we compute precision, and top-k accuracy to further validate the performance of Clone-Advisor on unseen dataset.

#### Experimental results

**RQ1: Naturalness **To assess how the original output of DeepClone model differs from the real clone code methods, we find the perplexity scores of the clone method predicted by DeepClone and top-k most similar retrieved clone methods. Table 6 depicts the mean perplexities of top-ten Clone-Advisor recommendations, DeepClone output, and ground truth (GT). We observe quite high mean perplexity scores and standard deviation for the DeepClone output (11.624 *±* 5.892). This indicates high *noise* and less natural code, which is a known problem of neural language generation (Li et al., 2017a; Shao et al., 2017). However, we notice quite low mean perplexity scores and standard deviation for the ground truth (2.047 *±* 0.848) and top-ten Clone-Advisor recommendations (range from 1.79 *±* 0.716 till 1.907 *±* 0.612) against the set of 735 input queries. This depicts that the top-ten retrieved snippets have relatively low perplexities, which indicates that they are highly natural and less noisy as compared to DeepClone output. There are slight variations in the perplexity values of Top-ten samples, which can be attributed to various factors such as the type of functionality, the number of clone method snippet trained in the DeepClone model, and inner similarity among the clone methods’ type. These factors have been discussed in detail in our previous work (Hammad et al., 2020a). These numbers show that the IR technique can improve the predicted clone method generated by DeepClone, resulting in more natural snippets.

Clone-Advisor produces more natural output compared to DeepClone.

**Table 6:** Mean perplexities related to different clone method outputs.

	Perplexity
DCO	11.624 *±* 5.892
GT	2.046 *±* 0.848
1	1.785 *±* 0.712
2	1.841 *±* 0.712
3	1.796 *±* 0.602
4	1.819 *±* 0.536
5	1.875 *±* 0.546
6	1.9 *±* 0.579
7	1.883 *±* 0.563
8	1.869 *±* 0.528
9	1.828 *±* 0.486
10	1.867 *±* 0.581

**RQ2: Exact Match Evaluation **We collect the top ten Clone-Advisor recommendations retrieved by our approach, and compute the top-k accuracy (the fraction of times the ground truth clone method appears in the top-k Clone-Advisor recommendations) for k ∈ [1, 10]. Moreover, we measure the Mean Reciprocal Rank (MRR) scores for the recommendations. A simplified description of MRR is that it averages top-k accuracy across various k. In this specific scenario k ∈ [1, 10] since the methodology output a list of top-ten Clone-Advisor recommendations. The MRR is a rank-based evaluation metric, which produces a value between 0 and 1, where a value closer to one indicates a better clone method recommendation system. The reciprocal rank of a query response is the multiplicative inverse of the rank of the first correct answer, while MRR is the average of reciprocal ranks. We have further calculated top-k accuracy and MRR involving an exact match of the Clone-Advisor recommendations with the ground truth. We achieve an accuracy of 39.3% in the top-ten Clone-Advisor recommendations and MRR as 28.3% (see Table 7). In a fair share of the cases, we can find exactly the same clone method as in the ground truth.

Clone-Advisor can generate recommendations which exactly match with the ground truth up to 40.5% top-ten accuracy.

**Table 7:** Evaluation results between ground truth and top 10 recommended clones in terms of MRR and Top-k accuracies (‘*’ = Overall score, ‘°’ = method name exists in the context, ‘!’ = method name does not exist in the context).

	MRR	Top-1	Top-3	Top-5	Top-10
**Exact match**					
*	0.290	0.238	0.325	0.362	0.405
∘	0.301	0.245	0.340	0.380	0.429
**!**	0.167	0.157	0.186	0.186	0.186
**Functionality match**					
*	0.740	0.694	0.770	0.801	0.845
∘	0.764	0.719	0.791	0.821	0.865
**!**	0.516	0.457	0.571	0.614	0.657

**RQ3: Functionality Match Evaluation **As BigCloneBench contains references to various implementations for each of the 43 functionalities, it is quite possible that the user is recommended a different snippet than the ground truth, yet implementing the same functionality. This alternative can also help the developer achieve their goal. To assess such cases, we have calculated the top-k accuracy and MRR taking alternative implementations into account. In this case, we achieve quite a high accuracy, notably 84.1% in the top-ten Clone-Advisor recommendations, as well as 73.8% MRR in terms of functionality match with the ground truth (see Table 5). The results display that our methodology has the capability of identifying a good match between ground truth and top-k Clone-Advisor recommendations. Table S3 displays that top-one recommended clone method exactly matches the ground truth (see Table 5). This is a major improvement over the exact match scores and further reinforces the claims we make for our approach.

Clone-Advisor can produce recommendations, whose functionalities match with the ground truth up to 84.5% top-ten accuracy.

**RQ4: Multiple Functionality Match Evaluation **We collect the top ten Clone-Advisor recommendations retrieved by our approach, and compute Precision@k (Table 8) with various values for k. The columns P@1, P@3, P@5 and P@10 show the results of the average Precision@k over all queries when k is 1, 5 and 10, respectively. In this case, we observe quite a high precision score, notably 69.4% in the top-one Clone-Advisor recommendations. The results display that our methodology has the capability of identifying multiple matches between ground truth and top-k Clone-Advisor recommendations.

Clone-Advisor can produce more than one correct recommendation, whose functionality matches with the ground truth up-to 69.4% P@1 and 57.8% P@10 precision.

**Table 8:** Evaluation results between ground truth and top 10 recommended clones in terms of precision (P@k) (‘*’ = Overall score, ‘°’ = method name exists in the context, ‘!’ = method name does not exist in the context).

	P@1	P@3	P@5	P@10
*	0.694	0.656	0.636	0.578
∘	0.719	0.676	0.655	0.595
**!**	0.457	0.471	0.46	0.417

**RQ5: Validation on Other Datasets **Many researchers build several benchmarks to evaluate their code search techniques based on natural language queries such as Lv et al. (2015), Kim et al. (2018), Gu, Zhang & Kim (2018) and Cambronero et al. (2019). We follow a similar idea and create our own benchmark by collecting code samples belonging to each functionality type existing in BigCloneBench from various websites such as Stack Overflow (https://stackoverflow.com/), and ProgramCreek (https://www.programcreek.com/). We search for the description mentioned against each functionality type in the BigCloneBench corpus using Google search engine. Once web-page lists are retrieved, we manually analyze code samples available in those pages by considering the following criteria:

1. Sample should be a clone of the searched functionality type.
2. Sample should belong to the Java programming language.
3. Sample should have a method signature along with a complete method body.
4. If sample is found from Stack Overflow website, we ensure that either the answer containing that sample has positive votes or has an acceptance status.

We apply preprocessing steps such as marking, normalization, and replacement as mentioned in “Dataset preparation”. Our task is to complete the clone method body based on the input context, and recommend real clone methods. We take first 20 tokens as the input and pass it to our DeepClone model to generate clone method. We pass the generated clone method to Clone-Advisor to get the recommended clone methods. We calculate MRR, top-k accuracy and precision by inspecting functionality types of top-k recommended clone methods and ground truth (Tables 9 and 10). Our benchmark is publicly accessible from our website (https://www.win.tue.nl/~mhammad/Clone-Advisor/cloneadvisor.html). We can see from the results that we can find the required clone method among top@5 recommended methods for all the code samples. We further note down that for functionality types 5 and 9, most of the recommended clone methods do not belong to the functionality type of the ground truth. This is because clone methods of these functionality types exist few in numbers in BigCloneBench corpus, which effects the performance of DeepClone model to not perfectly learn their patterns. As a result, DeepClone model generates buggy a method body and Clone-Advisor is also not perfectly be able to find similar clone methods of these functionality types. Though, these code samples do not represent the clones of all functionality types which exist in the BigCloneBench dataset, but at least it gives an impression that our methodology can work on unseen code samples.

Clone-Advisor can produce more than one correct recommendation, whose functionality type matches with the ground truth on other datasets.

**Table 9:** Evaluation results between ground truth and top 10 recommended clones in terms of MRR and top-k accuracies for code samples available in our own benchmark.

ID	MRR	Top-1	Top-3	Top-5	Top-10
2	1	1	1	1	1
3	1	1	1	1	1
4	1	1	1	1	1
5	0.333	0	1	1	1
6	1	1	1	1	1
7	1	1	1	1	1
8	1	1	1	1	1
9	0.25	0	0	1	1
10	1	1	1	1	1
11	1	1	1	1	1
12	1	1	1	1	1
13	1	1	1	1	1
14	1	1	1	1	1
15	1	1	1	1	1
17	1	1	1	1	1
18	1	1	1	1	1
19	0.333	0	1	1	1
20	1	1	1	1	1
21	1	1	1	1	1
22	0.5	0	1	1	1
23	1	1	1	1	1
24	1	1	1	1	1
25	1	1	1	1	1
26	1	1	1	1	1
27	1	1	1	1	1
28	1	1	1	1	1
29	1	1	1	1	1
30	0.333	0	1	1	1
31	0.5	0	1	1	1
32	0.25	0	0	1	1
33	1	1	1	1	1
34	1	1	1	1	1
35	1	1	1	1	1
36	1	1	1	1	1
37	1	1	1	1	1
38	1	1	1	1	1
39	1	1	1	1	1
40	1	1	1	1	1
41	1	1	1	1	1
42	1	1	1	1	1
43	1	1	1	1	1
44	1	1	1	1	1
45	1	1	1	1	1
Average	0.895	0.837	0.953	1	1

**Table 10:** Evaluation results between ground truth and top 10 recommended clones in terms of precision (P@k) for code samples available in our own benchmark.

ID	P@1	P@3	P@5	P@10
2	1	1	1	0.9
3	1	1	1	1
4	1	1	1	1
5	0	0.333	0.2	0.1
6	1	1	1	1
7	1	0.333	0.4	0.5
8	1	0.667	0.4	0.4
9	0	0	0.2	0.1
10	1	1	0.8	0.4
11	1	1	0.8	0.4
12	1	1	1	0.7
13	1	1	1	0.9
14	1	1	1	1
15	1	1	1	0.8
17	1	0.667	0.6	0.6
18	1	0.667	0.6	0.5
19	0	0.333	0.4	0.7
20	1	1	0.6	0.3
21	1	1	1	0.6
22	0	0.333	0.4	0.6
23	1	1	1	1
24	1	1	1	1
25	1	0.667	0.4	0.3
26	1	1	0.8	0.5
27	1	1	1	1
28	1	1	1	1
29	1	0.667	0.4	0.4
30	0	0.333	0.4	0.5
31	0	0.333	0.2	0.4
32	0	0	0.4	0.6
33	1	0.667	0.8	0.9
34	1	0.667	0.6	0.4
35	1	1	0.8	0.9
36	1	0.667	0.4	0.4
37	1	0.333	0.2	0.3
38	1	1	0.8	0.5
39	1	0.333	0.4	0.3
40	1	0.667	0.4	0.2
41	1	1	1	1
42	1	0.667	0.6	0.5
43	1	0.333	0.2	0.2
44	1	0.667	0.6	0.4
45	1	1	0.6	0.3
Average	0.840	0.730	0.600	0.590

## Discussion

### Usefulness of Clone-Advisor recommendations

In our qualitative investigation, we experienced two different scenarios based on the recommended output. The first one is the ideal scenario when one of the top-k Clone-Advisor recommendations exactly match the ground truth. In the example given in Table S3, “transpose” at top-1 exactly matches the ground truth. This scenario gives the best results. The second scenario is when none of the top-k recommended methods exactly match the ground truth but at least one of the top-k recommended clone method functionality matches with functionality of the ground truth. In Table S3, although top-1 and top-2 Clone-Advisor recommendations do not exactly match with the ground truth, they belong to the same functionality “copy file”. The main advantage of our methodology is that even if the Clone-Advisor recommendations do not exactly match the ground truth, still they would be usually implementing the same functionality as the ground truth method, and might satisfy the user’s need.

Similarly, the performance of Clone-Advisor is highly dependent on input context. There are two scenarios based on the input context. The first scenario is when the context contains the name of the method. It is straightforward for the neural language technique to generate the predicted clone method following the given method name and current context. Table S3 gives an example of this scenario, where “transpose” method name is mentioned in the context and our approach recommends clone methods as top-one and top-two, whose functionality matches with the functionality of the ground truth. The second scenario is based on the context that does not contain a method name. This can have two different output sub-scenarios. The first one is when the functionality of the recommended clone method and the ground truth do not match. As we see in Table S4, the context does not have the full signature of the clone method. This makes the predicted output by DeepClone using nucleus sampling deviate from the functionality of the ground truth. Ground truth belongs to “copy file” functionality, while the DeepClone output belongs to “delete directory” functionality, which eventually leads to TF-IDF recommending clone methods as top-one and top-two around the functionality “delete directory”. The other output sub-scenario is when we manage to successfully generate the predicted clone method from DeepClone whose functionality matches with the ground truth. In Table S1, “copy file” method name is not mentioned in the context, but the functionality of the DeepClone output matches with the ground truth, which eventually helps TF-IDF to retrieve real clone methods on the basis of DeepClone output. We notice that the total number of ’’copy file’’ clone methods used in DeepClone training are 2,454, which allows nucleus sampling to predict the clone method from DeepClone closer to ground truth in example 1.

Such scenarios eventually affect evaluation measures involving exact matches, functionality matches and precision (see Tables 7 and 8). We manually annotated 735 contexts and identified 665 contexts, in which method name exists and 70 contexts in which method name does not exist. These are symbolically represented as ‘∘’ and ‘!’ in Tables 7 and 8. The results clearly depict that Clone-Advisor produces better recommendations, when the clone method name is included in the context.

Overall we believe our approach yields very promising results and can assist the developers by recommending real and accurate clone methods. We cannot empirically compare our methodology with Lancer on the clone completion task. This is because the dataset of 2,892 programming tasks used in Lancer is not publicly available and our requests to the authors did not receive any response.

### Limitations and threats to validity

Clone-Advisor is a major improvement over the DeepClone, and leads to promising results for recommending meaningful clone snippets. However, it has certain limitations as well. In our study, we relied on the HuggingFace transformer implementation of GPT-2 to train and evaluate DeepClone model. While GPT-2 is a reliable architecture that has been used in a number of NLP experiments (Radford et al., 2019b), HuggingFace transformer implementation is still an emerging project. However, our results and trends are aligned with those that have been obtained in the field of NLP. Hence, we are positive that the results are reliable. Another point is that we have selected and used various parameter/threshold values and techniques with the goal of showcasing the feasibility of our approach. As an example, for predicting clone methods, we only used nucleus sampling with threshold value of 0.95 (Holtzman et al., 2019). There are various other text generation methods such as beam search (Vijayakumar et al., 2018), sampling with temperature (Ackley, Hinton & Sejnowski, 1985), and top-k sampling (Fan, Lewis & Dauphin, 2018), which can be explored for predicting clone methods on the basis of user context. Similarly, threshold values can be tuned to get the best results.

As can be expected from a DNN-based study, we could not evaluate all the possible combinations (hundreds) of hyper-parameter due to the resources needed. There is a risk in the choice of hyper-parameters for deep learning methods. The change in training, validation or testing set or the variation in hyper-parameters may impact the performance of the anticipated method. For this reason, we also did not evaluate other NLM architectures such as additional neural cache variants (Merity et al., 2016, Vinyals, Fortunato & Jaitly, 2015) or QRNNs (Bradbury et al., 2016).

Based on experimentation, we determined certain parameters (*e.g.*, 735 as the number of subsequences, 20 as the number of tokens per subsequence) aiming to demonstrate a preliminary evaluation of our methodology. However, by having different parameters, *e.g.*, having subsequences of different sized tokens, and using the complete set of queries, we could have different results.

Another limitation involves the normalization step we have performed. We have replaced integer, float, binary, and hexadecimal constant values with the $\langle$num_val $\rangle$ meta-token. Similarly, we have replaced string and character values with $\langle$str_val $\rangle$. This reduces our vocabulary size, which leads to faster training of the model, but also reduces the vocabulary of the predictions. We nevertheless note that technique has been used by several researchers in the same manner for data preparation (White et al., 2015; Karampatsis et al., 2020). Similarly, in order to have fair comparison between the DeepClone output and the real clone methods available in search corpus, we have built a search corpus in the same format as we have used for DeepClone. This helps the TF-IDF technique to recommend clone methods accordingly. In the future, we plan to replace these meta tokens with original constant values of real clone methods, so that these clone methods can work in Java based IDE tools such as Eclipse, without leading to syntax errors. Moreover, soc and eoc tokens help nucleus sampling to predict clone method from DeepClone model. Same meta tokens have been used in search corpus to help TF-IDF to have fair enough Clone-Advisor recommendations. In the future, we aim to remove these meta-tokens, so that these Clone-Advisor recommendations can work directly in IDEs. In this study, we only apply TF-IDF for retrieving the most similar real clone methods, based on the DeepClone prediction. However, there are other IR techniques such as GLOVE (Pennington, Socher & Manning, 2014), and word2vec (Mikolov et al., 2013), which can be additionally explored. We leave it for future work to comparatively assess and optimize the parameters and techniques for our approach.

**Validation on Other Datasets **In this paper, we have introduced the fundamental techniques and evaluated them with respect to multiple aspects by focusing on BigCloneBench dataset. This gives a strong foundation on the feasibility of using our approach. However, there is a limitation, which originates from the selected dataset. Despite the fact that the dataset used in this study is collected from a well-known clone code dataset (BigCloneBench), it does not necessarily mean the codebase represents the Java language source code entirely. Similarly, BigCloneBench contains only references of 43 functionalities, which does not represent all types of functionalities existing in different publicly available datasets.

It is not appropriate, if we evaluate Clone-Advisor and DeepClone on other functionalities, which do not exist in BigCloneBench. This is because the DeepClone model has been currently trained and learned the patterns of only 43 different types of functionalities, which exist in BigCloneBench. To evaluate our proposed study on a completely new dataset, there are two options. One option is to obtain new datasets of source code by first detecting method-level code clones in the literature and thus getting explicit clone references. For this purpose, there are various clone detection tools available such as SourcererCC (Sajnani et al., 2016), NiCAD (Cordy & Roy, 2011) and Clone Miner (Basit & Jarzabek, 2009) that can detect various types of clones with high precision.

The second option is to conduct our study on available clone related datasets, which can be used for clone completion. Some of the popular datasets of a similar kind include Project CodeNet (PCN) (Puri et al., 2021), Pedagogical programming Open Judge (POJ-104) (Mou et al., 2016) and Google Code Jam(GCJ) (Ullah et al., 2019). PCN, POJ-104 and GCJ are mined through online judge websites. They contain various problems, and each problem contains submitted solutions of students in terms of whole file. So all those files belonging to some problem are syntactically or semantically equivalent to each other. These datasets do not contain non-clone parts, because the whole file is considered to be clone of other files. However, in IJaDataset, which is being referenced by BigCloneBench, files can be marked at clone method level and remaining code is considered to be a non-clone part. So, in order to perform experimentation on these kind of datasets, clones needs to be marked at the start and end of each solution file with meta tokens such as soc at the start, and eoc. Then, after performing preprocessing steps mentioned in “Dataset preparation”, there will be a need to first fine-tune GPT-2 model on them. This will make a model to first learn the patterns of new clones. Afterwards, there is a need to replace a search corpus with new dataset in Clone-Advisor. This will make Clone-Advisor to recommend clones similar to the buggy clone snippet generated by the model. We plan to comparatively evaluate our proposed approach on new datasets in the future.

## Conclusion and future work

In this work, we presented and elaborated DeepClone, a deep learning based cloned code language model. We have developed the fundamental techniques and evaluated them with respect to multiple aspects. We performed intrinsic and extrinsic evaluations to determine the performance of the DeepClone model in predicting clone methods. The extensive evaluation suggests that the proposed approach significantly improves code prediction by exploiting the concept of deep learning and code clones. Due to the probabilistic nature of DeepClone, however, the original prediction deviates from real clones and contains errors. In order to alleviate this, we presented a novel approach, Clone-Advisor, which is based on IR to recommend real clone methods. This approach significantly improves the original output of DeepClone. We performed quantitative evaluation using a wide range of metrics, and qualitatively discussed additional scenarios, to support our claim. Our approach overall yields promising results and can substantially help programmers to rapidly write code.

The proposed LM can be potentially improved by hyper-parameter optimization, as well as by better training (*e.g.*, on a larger dataset or larger pre-trained GPT-2 models). We also plan to investigate how to tackle different types and granularity levels of code clones such as simple clones, structural clones, file clones, and clones of other artifact types such as models (Babur, Cleophas & van den Brand, 2019; Hammad et al., 2020b; Hammad et al., 2021). Moreover, we plan to perform a comparative study by evaluating different IR techniques such as BERT; pretrained word embedding techniques such as word2vec (Mikolov et al., 2013) and GLOVE (Pennington, Socher & Manning, 2014); and code query formulation techniques (Lu et al., 2015, Nie et al., 2016). Moreover, we aim to develop and evaluate a visualization tool on top of our system to provide a user-friendly environment for assisting the developers. This also includes fully automatic formatting of the code rather than the semi-automatic approach we have taken in this paper. In future work, we also plan to build tool support and evaluate the effectiveness and usefulness of our approach in a real world setting with real developers.

## Supplemental Information

10.7717/peerj-cs.737/supp-1 Supplemental Information 1 Java Files.

10.7717/peerj-cs.737/supp-2 Supplemental Information 2 Tables S1–S4

## Additional Information and Declarations

### Data Availability

The following information was supplied regarding data availability: The code is available at: https://www.win.tue.nl/~mhammad/Clone-Advisor/cloneadvisor.html.
