# Supplementary material for: Clone-advisor: recommending code tokens and clone methods with deep learning and information retrieval
Source: PeerJ Comput Sci. 2021 Nov 9;7:e737. doi: 10.7717/peerj-cs.737 (PMC8641571; doi:10.7717/peerj-cs.737)
Supplement: Supplemental Information 2 [file peerj-cs-07-737-s002.pdf]

Table S1. An example containing scenarios such as functionality type match and method name not mentioned in the context

Context

```
substring(i + <num_val >).toLowerCase();
}
return ext ;
}
<soc> public static void
```

Ground truth (GT)

```
<soc> public static void copyFile ( File in , File out ) throws IOException {
    FileChannel inChannel = new FileInputStream(in).getChannel( );
    FileChannel outChannel = new FileOutputStream(out).getChannel( );
    try {
        inChannel.transferTo(<num_val>, inChannel.size(), outChannel);
        }
    catch ( IOException e ) {
        throw e ;
    }
    finally {
        if (inChannel != null) inChannel.close ( );
        if (outChannel != null) outChannel.close ( );
        }
} <eoc>
```

**Perplexity**	1.21

DeepClone output (DCO)

```
<soc> public static void copyStreams ( File source, File dest, File destDir ) throws IOException {
    BufferedInputStream in = null ;
    BufferedOutputStream out = null ;
    try {
        in = new BufferedInputStream ( new FileInputStream ( source ) ) ;
        out = new BufferedOutputStream ( new FileOutputStream ( dest ) ) ;
        out.write(source.getAbsolutePath( ));
        int len = in.read ( ) ;
        while(len > <num_val >){
            out.write(len);
        }
        in.close( );
        out.close( );
    }
    catch (Exception ex) {
        ex.printStackTrace ( ) ;
        }
    finally {
        if ( in!= null ) in.close( ) ;
        if ( out!= null ) out.close ( ) ;
        }
} <eoc>
```

**Perplexity**	9.246

Top 1

```
<soc> public static void copy ( File source , File dest ) throws Exception {
    FileInputStream in = new FileInputStream ( source );
    FileOutputStream out = new FileOutputStream ( dest ) ;
    int c ;
        while((c = in.read()) != - <num_val >)
            out.write(c);
            in.close();
            out.close();
    }
<eoc>
```

**Perplexity**	1.439

Top 2

```
<soc> public static void copyFile ( String source , String dest ) throws IOException {
    FileChannel in = null , out = null ;
    try {
        in = new FileInputStream ( new File ( source ) ) . getChannel ( ) ;
        out = new FileOutputStream ( new File ( dest ) ) . getChannel ( ) ;
        in . transferTo ( <num_val> , in . size ( ) , out ) ;
        }
    finally {
        if (in!=null) in.close();
        if (out!=null) out.close();
    }
} <eoc>
```

**Perplexity**	1.321

Table S2. An example of the preprocessing steps applied on a clone method

Original

```
//Heuristic: (([0-9,a-z,A-Z,_,$]+\s*\=\s*\(\s*[0-9,a-z,A-Z,_,$]+\s*\+\s*[0-9,a-z,A-Z,_,$]
 // imid = imin + (imax - imin)/2;
 // imid = (imax + imin)/2;
 public static int binarySearch1(int arr[], int key, int imin, int imax) {
     //Implementation: Recursive, primitive type
     if (imax < imin)
         return -1;
     int imid = (imin + imax) / 2;
     if (arr[imid] > key)
         return binarySearch1(arr, key, imin, imid - 1);
     else if (arr[imid] < key)
         return binarySearch1(arr, key, imid + 1, imax);
     else
         return imid;
 }
```

Marking

```
//Heuristic: (([0-9,a-z,A-Z,_,$]+\s*\=\s*\(\s*[0-9,a-z,A-Z,_,$]+\s*\+\s*[0-9,a-z,A-Z,_,$]
 // imid = imin + (imax - imin)/2;
 // imid = (imax + imin)/2;
<soc> public static int binarySearch1(int arr[], int key, int imin, int imax) {
     //Implementation: Recursive, primitive type
     if (imax < imin)
         return -1;
     int imid = (imin + imax) / 2;
     if (arr[imid] > key)
         return binarySearch1(arr, key, imin, imid - 1);
     else if (arr[imid] < key)
         return binarySearch1(arr, key, imid + 1, imax);
     else
         return imid;
 } <eoc>
```

Normalization

```
<soc> public static int binarySearch1 ( int arr[], int key, int imin, int imax ) {
 if ( imax < imin ) return -1 ; int imid = ( imin + imax ) / 2 ;
 if ( arr [ imid ] > key ) return binarySearch1 ( arr , key , imin , imid - 1 ) ;
 else if ( arr [ imid ] < key ) return binarySearch1 ( arr , key , imid + 1 , imax ) ;
 else return imid ; } <eoc>
```

Replacement

```
<soc> public static int binarySearch1 ( int arr[], int key, int imin, int imax ) {
 if ( imax < imin ) return <num_val> ; int imid = ( imin + imax ) / <num_val> ;
 if ( arr [ imid ] > key ) return binarySearch1 ( arr , key , imin , imid - <num_val> ) ;
 else if ( arr [ imid ] < key ) return binarySearch1 ( arr , key , imid + <num_val> , imax ) ;
 else return imid ; } <eoc>
```

Table S3. An example containing scenarios such as exact match, functionality type match and method name mentioned in the context

Context

```
v[i][<num_val>];
}
<soc> public static Rectangle2D[][] transpose(Rectangle2D
```

Ground truth (GT)

```
<soc> public static Rectangle2D[][] transpose(Rectangle2D [][] a) {
        int m=a.length;
        int n=a[<num_val>].length;
        Rectangle2D [][] t = new Rectangle2D[n][m];
        for(int i=<num_val>; i < m; i++)
            for (int j = <num_val>; j < n;j++)
                t[j][i]=a[i][j];
                return t;
    } <eoc>
```

Perplexity 1.541

DeepClone output (DCO)

```
<soc> public static Rectangle2D[][] transpose(Rectangle2D[][] a, Rectangle2D[][] b)
{
    int[][] M = new int[a.length][a[<num_val>].length];
    for(int i =<num_val>; i < m.length;i++) {
        for(int j = <num_val>; j < a[i].length;j++){
            M[j][j]=b[i][j];
            }
        }
    return M;
} <eoc>
```

Perplexity 3.54

Top 1

```
<soc> public static Rectangle2D[][] transpose(Rectangle2D[][] a){
    int m = a.length;
    int n = a[<num_val>].length;
    Rectangle2D[][] t=new Rectangle2D[n][m];
    for (int i = <num_val>;i<m; i++)
        for (int j=<num_val>;j<n;j++)
            t[j][i]=a[i][j];
    return t;
} <eoc>
```

Perplexity 1.541

Top 2

```
<soc> public static byte[][] transpose(byte [][] m) {
    byte[][] n = new byte[m[<num_val>].length][m.length];
    for (int j = <num_val>;j<m.length;j++)
        for (int i = <num_val>;i<m[<num_val>].length;i++)
            n[i][j]=m[j][i];
    return n;
} <eoc>
```

Perplexity 1.63

Table S4. An example containing scenarios such as functionality type not matched and method name not mentioned in the context

Context

```
MAX_IO_CHUNK_SIZE = <num_val> * <num_val> * <num_val> ;
private static final long LASTMODIFIED_DIFF_MILLIS = <num_val> ;
<soc> public static boolean
```

Ground truth (GT)

```
<soc> public static boolean copy(File from, File to, Override override) throws IOException {
    FileInputStream in = null;
    FileOutputStream out = null;
    FileChannel srcChannel = null;
    FileChannel destChannel = null;
    if (override == null) override = Override.NEWER;
    switch (override) {
        case NEVER:
            if (to.isFile())
                return <num_val> ;
            break;
        case NEWER:
            if (to.isFile() && (from.lastModified() - LASTMODIFIED_DIFF_MILLIS) < to.lastModified())
                return <num_val> ;
            break;
    }
    to.getParentFile().mkdirs();
    try { in = new FileInputStream(from);
        out = new FileOutputStream(to);
        srcChannel = in .getChannel();
        destChannel = out.getChannel();
        long position = <num_val> ;
        long count = srcChannel.size();
        while (position < count) {
            long chunk = Math.min(MAX_IO_CHUNK_SIZE, count - position);
            position += destChannel.transferFrom(srcChannel, position, chunk);
        }
        to.setLastModified(from.lastModified());
        return <num_val> ;
    } finally {
        CommonUtils.close(srcChannel);
        CommonUtils.close(destChannel);
        CommonUtils.close(out);
        CommonUtils.close( in );
    }
} <eoc>
```

Perplexity	3.109

DeepClone output (DCO)

```
<soc> public static boolean deleteDirectory(File f) {
    try {
        final File[] fFiles = f.listFiles();
        for (int i = <num_val> ; i < fFiles.length; i++) {
            if (fFiles[i].isDirectory())
                deleteDirectory(fFiles[i]);

        }
    } catch (Exception e) {
        System.out.println( <str_val> +f + <str_val> +i + <str_val> );
        return <num_val> ;
    }
    return <num_val> ;
} <eoc>
```

Perplexity	6.137

Top 1

```
<soc> private void deleteDirectory ( File dir ) {
    File [ ] a = dir . listFiles ( ) ;
    if ( a != null ) {
        for ( File f : a ) {
            if (f.isDirectory()) {
                deleteDirectory ( f ) ;
            } else {
                f.delete( );
            }
        }
    }
    dir.delete ( );
    }
<eoc>
```

Perplexity	1.887

Top 2

```
<soc> public static void deleteDirectory ( File dir ) {
    File [ ] files = dir . listFiles ( ) ;
    for ( File f : files ) {
        if ( f . isDirectory ( ) ) {
            deleteDirectory ( f ) ;
        } else
            f.delete ( ) ;
    }
    dir.delete();
} <eoc>
```

Perplexity	1.831
